# Deep Illumina-Based Shotgun Sequencing Reveals Dietary Effects on the Structure and Function of the Fecal Microbiome of Growing Kittens

**DOI:** 10.1371/journal.pone.0101021

**Published:** 2014-07-10

**Authors:** Oliver Deusch, Ciaran O’Flynn, Alison Colyer, Penelope Morris, David Allaway, Paul G. Jones, Kelly S. Swanson

**Affiliations:** 1 WALTHAM Centre for Pet Nutrition, Waltham-on-the-Wolds, Leicestershire, United Kingdom; 2 Department of Animal Sciences, University of Illinois, Urbana, Illinois, United States of America; 3 Division of Nutritional Sciences, University of Illinois, Urbana, Illinois, United States of America; 4 Department of Veterinary Clinical Medicine, University of Illinois, Urbana, Illinois, United States of America; Hospital for Sick Children, Canada

## Abstract

**Background:**

Previously, we demonstrated that dietary protein:carbohydrate ratio dramatically affects the fecal microbial taxonomic structure of kittens using targeted 16S gene sequencing. The present study, using the same fecal samples, applied deep Illumina shotgun sequencing to identify the diet-associated functional potential and analyze taxonomic changes of the feline fecal microbiome.

**Methodology & Principal Findings:**

Fecal samples from kittens fed one of two diets differing in protein and carbohydrate content (high–protein, low–carbohydrate, HPLC; and moderate-protein, moderate-carbohydrate, MPMC) were collected at 8, 12 and 16 weeks of age (n = 6 per group). A total of 345.3 gigabases of sequence were generated from 36 samples, with 99.75% of annotated sequences identified as bacterial. At the genus level, 26% and 39% of reads were annotated for HPLC- and MPMC-fed kittens, with HPLC-fed cats showing greater species richness and microbial diversity. Two phyla, ten families and fifteen genera were responsible for more than 80% of the sequences at each taxonomic level for both diet groups, consistent with the previous taxonomic study. Significantly different abundances between diet groups were observed for 324 genera (56% of all genera identified) demonstrating widespread diet-induced changes in microbial taxonomic structure. Diversity was not affected over time. Functional analysis identified 2,013 putative enzyme function groups were different (*p*<0.000007) between the two dietary groups and were associated to 194 pathways, which formed five discrete clusters based on average relative abundance. Of those, ten contained more (*p*<0.022) enzyme functions with significant diet effects than expected by chance. Six pathways were related to amino acid biosynthesis and metabolism linking changes in dietary protein with functional differences of the gut microbiome.

**Conclusions:**

These data indicate that feline feces-derived microbiomes have large structural and functional differences relating to the dietary protein:carbohydrate ratio and highlight the impact of diet early in life.

## Introduction

Animals co-evolved with the microbiota that inhabit their guts, with direct host-microbiota interactions and microbe-microbe interactions that may impact the host indirectly [1]. The subsequent relationship between the host and the microbiota is complex and has an impact across a broad range of physiology that may impact health maintenance and disease progression [2], [3]. The importance of the microbiota in health and disease is well established in humans, with studies highlighting a wide range of associations within the immune system (such as autoimmunity and allergy), metabolism (metabolic syndrome [4], obesity [5] and diabetes [6]), disease (periodontitis [7]) as well as cognition (depression [8], anxiety [9] and autism [10]).

Characterization of the gut microbiota and its surrogate, the fecal microbiota, has identified different ecotypes and taxonomic structures associated with ethnicity, diet and genetics as well as health status in humans [11]–[13]. Furthermore, dietary interventions such as probiotics and prebiotics have been suggested as mechanisms to manipulate microbiota structure and microbiome function [14], [15].

Characterization of the gut microbiota has undergone many developments over recent decades. Much early work was focused on the enumeration and isolation and identification of bacteria to characterize their physiology in vitro. The development of DNA-based techniques allowed microbial profiling by DGGE and hypothesis-driven studies quantifying bacteria of interest using 16S rRNA primers. With the advent of next generation sequencing (NGS) and the greater availability of bioinformatics tools, detailed 16S rRNA classification of the gut microbiota could be generated. To transition from observation to interpretation of these more complex datasets, there is an increased requirement to use bioinformatics approaches to analyze data and to translate it into forms suitable for biological interpretation. The functional potential of a microbiome may be hypothesized from sequenced bacterial genomes. However, the development of NGS tools and bioinformatics databases to enable non-targeted sequencing of the genome has provided an opportunity to undertake global analysis of the functional potential of the whole microbiome and to develop a better understanding of the gut microbial ecosystem. Through a better understanding of the functional potential of different microbial structures, it may be possible to gain insights into how they establish, how host health-associated populations can be sustained and how best to reduce the load of potentially pathogenic bacteria through various life stages.

Careful study designs enable the transition from characterization and observation to hypothesis generation. One approach to support better interpretation of large microbiome datasets is to use genetically-related individuals, housed together in a similar environment from an early age that consume nutritionally complete diets over a period of time. Such conditions should support the investigation of nutritional interventions on the gut microbiota.

One group of individuals that provide a unique opportunity to investigate the effect of diet on the microbiome is companion animals because they are fed specific diets. Responsible pet food companies provide nutritionally complete food available from weaning and such diets may be fed for the entire lifetime of these individuals. Furthermore, the nutritional composition may influence the development and stability of the gut microbiome. One companion animal species of interest is the cat, which despite adaptations to being an obligate carnivore, can be fed a wide range of macronutrients (i.e. protein, fat and carbohydrate) within existing commercial cat diets. There is little known about the feline gut microbiome and how it is impacted by diet. Previously, a 16S rRNA sequencing project in cats identified that diet (protein: carbohydrate ratio) can influence the taxonomic distribution of the fecal microbiota [16]. Kittens were fed a high-protein, low-carbohydrate (HPLC) or a moderate-protein, moderate-carbohydrate (MPMC) diet from weaning. Fecal samples collected at 8, 12 and 16 weeks of age identified bacterial species with statistically significant differences in their abundances between the two diets, especially within the phyla Firmicutes, Actinobacteria and Fusobacteria. However, in that study it was not possible to explore potential functional aspects of these taxonomic differences. To progress from taxonomic characterization to functional interpretation we have used the same fecal samples from these kittens to analyze them using Illumina shotgun sequencing. Homology searches of sequenced short random DNA fragments against a database of known genes enables insights into microbial taxonomy and gene functions encoded in the metagenome of a microbiome. Protein coding genes can subsequently be assigned to ortholog groups (set of genes with the same putative enzyme function in different genomes inferred based on amino acid sequence similarity) and mapped to reference pathways such as KEGG [17] or SEED [18] to classify enzyme function (e.g. noted as KEGG level four) by biochemical pathways (noted as KEGG level three).

The purpose of this current study was to interpret shotgun data derived from deep Illumina shotgun sequencing of cat fecal samples and to determine whether functional attributes that were found to be most significant could be interpreted within the context of a diet intervention study.

## Results

### Annotating 345 Gb of feline gut microbiome data

DNA originally isolated from 36 kitten fecal samples for 16S amplicon analysis by Hooda et. al [16] was shotgun sequenced on the Hi-Seq Illumina platform for metagenomics analysis. A total of 345.3 gigabases (Gb) of sequence was generated, resulting in an average of 95.9 million reads (range = 63.6 to 136.2 million reads) per sample. Less than one percent of sequence reads mapped to the cat genome (data not shown) thus no filtering for host sequences was performed. Short reads were trimmed and compared against a reference database of 5,488,681 genes from 1,691 fully-sequenced genomes (1,685 bacteria, 4 archaea and 2 viruses) using the composition vector approach implemented in MetaCV [19]. 32% and 19% of reads were annotated to the genus taxonomic level and to KEGG ortholog groups for functional inference, respectively (**Table S1**).

Overall, 99.75% of all annotated reads were identified as coming from bacteria, with approximately 0.25% and less than 0.01% as archeal and viral, respectively. At the genus level, 26% and 39% of sequence reads were annotated for kittens fed the HPLC and MPMC diet, respectively. Functional annotation (KEGG; approximately 2.4 million sequences in database at time of annotation) was lower for samples collected from kittens fed the HPLC diet (16% of sequence reads) compared to those fed the MPMC diet (22% of sequence reads, **Table S1**). Although the percentage of annotated reads was different between diet groups, this difference was consistent over time (e.g., week 8, 12, 16).

### Characterization of taxonomic structure

Overall, 32 phyla, 230 families, 578 genera and 1,114 species of prokaryotes were identified in kitten feces. All taxonomic annotations for superkingdom, phylum, class, order, family, genus and species are provided in **Table S2**. Although many taxa were identified at each level of the NCBI taxonomy hierarchy, only a few taxa dominated. For example, 2 phyla, 10 families, 15 genera and 30 species contributed to more than 80 percent of the sequences at the respective taxonomic levels. Conversely, a lot of rare taxa contributed only few sequences each. In total, 23, 116, 241 and 525 taxa contributed less than one percent of the sequences at these same taxonomic levels.

**Figure 1:**
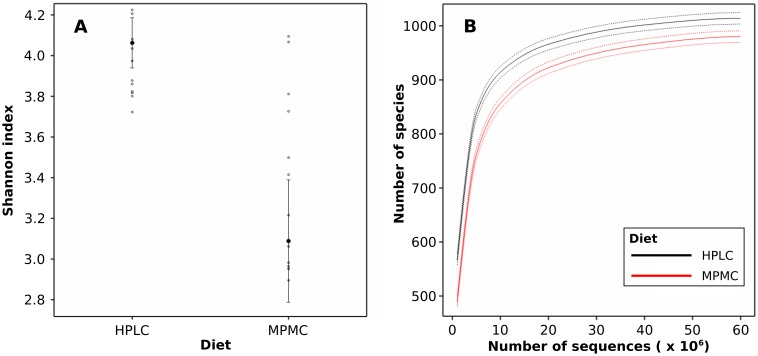
Species diversity and richness of feline fecal microbiomes by diet. (a) Shannon diversity indices as inferred from normalized species counts. (b) Rarefaction analysis of species richness. Species diversity and richness was significantly higher (with *p*<0.001) in the high-protein low-carbohydrate (HPLC) microbiome compared to the moderate-protein moderate-carbohydrate (MPMC) microbiome.

Species diversity estimates indicated a significantly increased (with *p*<0.001) fecal microbial diversity in kittens fed HPLC compared to MPMC, with average Shannon indices of 4.1 and 3.1, respectively (**Figure 1a** **, Table S3**). The residual variability was significantly smaller on the HPLC diet, after taking into account the between kitten variability, by likelihood ratio tests, *p*<0.001. No statistically significant differences in Shannon diversity were identified over time for kittens fed either diet (with *p*<0.05, **Table S3**). A rarefaction analysis also indicated higher species richness and diversity in HPLC compared to MPMC, as the respective curve reaches an earlier and lower plateau (**Figure 1b**). The curves were modeled and a subsequent statistical analysis identified significant differences between diet groups (with *p*<0.001, **Table S3**).

### Diet significantly affected microbial abundances

The aim of this study was to identify significant differences in taxa and the functional potential of the microbiome between diet groups and/or over time. Statistical analysis of all 578 genera and 230 families present in feline feces identified 324 genera and 138 families that were significantly different between diets, as assessed by generalized estimating equations (with *p*<0.00009 and 0.00022 respectively using a Sidak adjustment level to maintain an overall error rate of 5% per data set, see methods for details). Another five families and six genera had significant diet by time interactions. All genera and families with significant differences, p-values and 95% confidence intervals are listed in **Table S4**, with predominant bacterial genera presented in **Table 1**.

**Table 1:** Predominant bacterial genera (expressed as average percentage of sequences) in feces of kittens fed a moderate-protein, moderate-carbohydrate (MPMC) or high-protein, low-carbohydrate (HPLC) diet at 8, 12, and 16 weeks of age.

	Diets						
	MPMC			HPLC			
	Age (weeks)			Age (weeks)			
	8	12	16	8	12	16	Odds ratio (MPMC vs. HPLC)
**Firmicutes**	**53.84**	**57.34**	**61.78**	**45.98**	**53.63**	**50.89**	
* Acidaminococcus*	6.70	5.94	7.63	0.78	0.82	0.98	12.81
* Bacillus*	0.30	0.24	0.27	0.51	0.48	0.49	0.76
* Butyrivibrio*	0.58	0.40	0.53	1.53	1.47	1.43	0.45
* Cellulosilyticum*	0.25	0.16	0.21	0.62	0.63	0.61	0.45
* Clostridium*	5.23	3.71	4.60	13.92	13.78	13.96	0.43
* Ethanoligenens*	0.38	0.18	0.21	0.94	0.82	0.83	0.38
* Eubacterium*	2.77	1.86	2.51	7.67	17.22	13.68	0.23
* Lactobacillus*	3.55	0.55	1.07	1.41	0.65	0.67	
* Megasphaera*	19.74	32.69	30.66	0.29	0.28	0.43	151.67
* Oscillibacter*	0.87	0.40	0.51	1.87	1.77	1.67	0.42
* Roseburia*	2.23	1.38	1.89	6.12	5.91	6.10	0.40
* Ruminococcus*	0.55	0.32	0.39	1.26	1.24	1.15	0.46
* Selenomonas*	6.16	6.24	7.52	0.59	0.60	0.65	19.00
* Streptococcus*	0.75	0.44	0.55	1.22	1.04	1.00	
**Bacteroidetes**	**30.36**	**21.32**	**17.07**	**36.02**	**31.59**	**33.24**	
* Bacteroides*	11.59	6.74	6.74	22.50	19.73	19.90	0.50
* Odoribacter*	0.55	0.33	0.30	1.17	1.05	1.03	0.46
* Parabacteroides*	1.52	0.58	0.54	5.40	2.68	1.85	0.32
* Prevotella*	15.38	12.68	8.71	5.65	7.04	9.14	2.22
* Tannerella*	0.30	0.19	0.17	0.44	0.37	0.44	
**Actinobacteria**	**10.89**	**17.70**	**17.30**	**6.57**	**4.15**	**5.29**	
* Atopobium*	0.40	0.42	0.52	0.46	0.28	0.37	1.76
* Bifidobacterium*	7.22	14.76	13.79	0.56	0.47	0.78	34.40
* Coriobacterium*	1.24	0.79	0.95	2.25	1.11	1.61	
* Eggerthella*	0.48	0.33	0.40	1.17	0.75	0.77	0.63
* Olsenella*	0.66	0.77	0.94	0.68	0.40	0.55	2.16
* Slackia*	0.27	0.20	0.24	0.56	0.41	0.42	0.73
**Proteobacteria**	**2.67**	**2.00**	**2.03**	**5.90**	**5.22**	**4.75**	
* Desulfovibrio*	0.34	0.11	0.09	1.21	1.32	0.93	0.18
* Escherichia*	0.14	0.13	0.03	1.32	0.67	0.05	
**Fusobacteria**	**0.21**	**0.15**	**0.18**	**2.11**	**2.11**	**2.36**	
* Fusobacterium*	0.06	0.04	0.05	0.98	1.00	1.13	0.06
* Ilyobacter*	0.05	0.03	0.04	0.84	0.84	0.95	0.07
**Sprirochaetes**	**0.78**	**0.54**	**0.62**	**1.48**	**1.44**	**1.47**	
* Treponema*	0.40	0.26	0.31	0.81	0.80	0.81	0.54

Odds ratios are provided for statistically significant diet differences.

**Figure 2:**
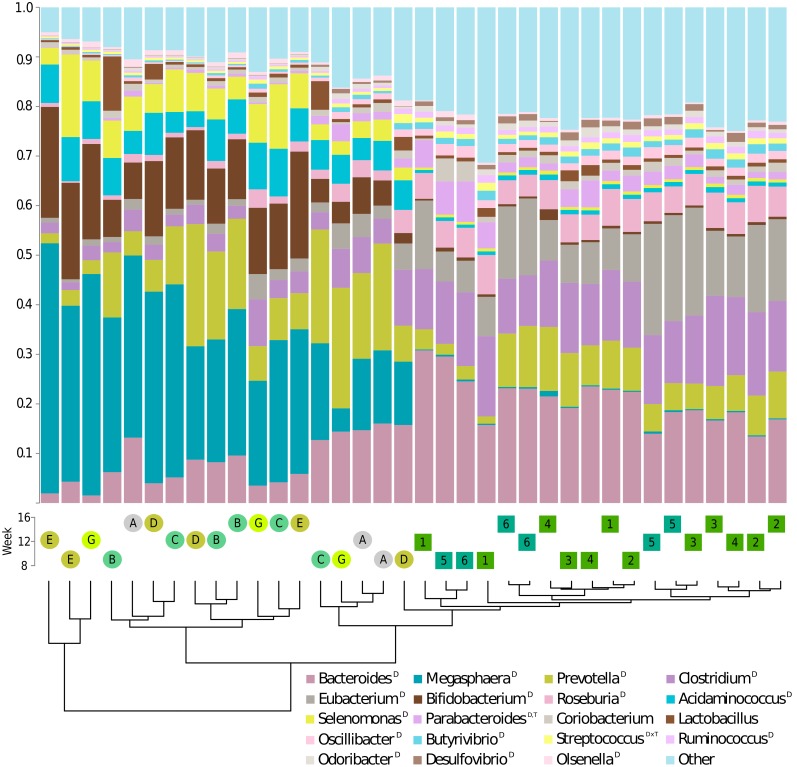
Taxonomic profiles for the 20 most abundant genera. The 19 most abundant genera were sorted from bottom to top by descending overall relative abundance in the 36 samples. The last interval summarizes all remaining genera. Hierarchical clustering of relative abundances of all genera clearly split samples by diet (see dendogram). Samples are coded by diet (rectangles/letters for high and circles/numbers for medium protein), litter (color) and week (vertical position). Superscript letters in legend indicate significant diet, time, or diet by time effects.

**Figure 2** illustrates taxonomic profiles of all 36 microbiome samples based on 20 bins; the 19 most abundant genera accounting for a mean of 83.4% of total reads, plus one bin summarizing the remaining 16.6% of reads for all other genera (**Table S2**). In kittens fed the MPMC diet, the top 19 genera accounted for a larger percentage compared to those fed HPLC (89.4% vs. 77.4% of annotated sequences, **Table S2**), illustrating a higher proportion of rare genera in the fecal microbiota of kittens fed the HPLC diet. The taxonomic profiles of all samples showed patterns characteristic for diet regardless of age. This was underlined by statistical analysis. Out of the 19 most prevalent genera, 16 had significantly different relative abundances between diets. One genus (*Streptococcus*) had a significant diet by time interaction. *Parabacteroides* was the only genus among the 16 genera significantly different by diet, which was also significantly different by time. Only 2 of the top 19 genera (*Coriobacterium* and *Lactobacillus*) had no statistically significant associations with diet or time. To visualize similarities between the 36 microbiota, a cluster analysis was performed on the relative abundances of all 578 genera (not only the 19 displayed). HPLC samples were grouped to the exclusion of all but one MPMC sample. Mean pair-wise Euclidean distances between samples were much smaller within the HPLC group (32,024) than within the MPMC group (108,468) indicating that the microbiota were more similar within the HPLC group.

### A higher number of taxa were more abundant in HPLC but those more abundant in MPMC had stronger ORs

Diet not only significantly affected the 19 most prevalent taxa shown in **Figure 2**, but also less prevalent taxa (**Table S4**). In total, of the 324 bacterial genera that were significantly different by diet, 30 were more common in MPMC (average odds ratio (OR) of 8.61) and 294 were more common in HPLC (average OR of 0.62). Twofold or higher differences were observed for 7 and 48 genera, respectively (OR> = 2.0 indicating odds twofold higher with MPMC and OR< = 0.5 indicating odds twofold lower with MPMC). Fourfold or bigger ORs were detected for *Megasphaera* (151.67), *Bifidobacterium* (34.40), *Selenomonas* (19.00), *Acidaminococcus* (12.81), *Eubacterium* (0.23), *Streptobacillus* (0.22), *Desulfovibrio* (0.18), *Ilyobacter* (0.07) and *Fusobacterium* (0.06).

Differences at the family level of bacterial taxonomy were very similar to those at the genus level (**Table S4**). Of the 138 families that were significantly different by diet, 12 were more common in MPMC (average OR of 10.06) and 126 were more common in HPLC (average OR of 0.65). Twofold or stronger differences were observed for 4 and 20 genera, respectively. Fourfold or bigger ORs were detected for *Veillonellaceae* (62.86), *Bifidobacteriaceae* (31.94), *Acidaminococcaceae* (12.81), *Eubacteriaceae* (0.24), *Desulfovibrioceae* (0.19) and *Fusobacteriaceae* (0.06).

### Over two thousand ortholog groups were significantly different by diet

In total, 2,182,187 unique protein-coding genes were identified in the microbiomes of feline feces (1,702,265 in HPLC and 1,440,712 in MPMC). Of those 1,217,213 (56%) had a KEGG annotation that was used to relate them to 6,660 KEGG ortholog groups (KEGG level 4; **Table S5**). The identified KEGG ortholog groups mapped to 280 biochemical pathways (KEGG level three groups). Similar to the taxonomy section, the distribution of individual ortholog groups was skewed. Only 10.1% of the ortholog groups contributed to more than 80% of the sequences, while 70.1% of ortholog groups contributed to less than 1%.

Statistical analyses using generalized estimating equations were carried out analogously to the taxonomy section with adjustments for multiplicity to identify ortholog groups with significant diet and time effects (with *p*<0.000007). Significant differences between kittens fed MPMC and those fed HPLC were observed for 2,013 unique groups contributing to 194 pathways (**Table S4**). Significant diet by time interactions were observed for 129 unique ortholog groups contributing to 66 KEGG pathways.

Of the 2,013 ortholog groups that were significantly different by diet, 1,183 had increased ORs with MPMC (average OR of 11.22) and 830 had increased ORs in the direction of HPLC (average OR of 0.32). This is similar to the taxonomy results whereby changes in the direction of MPMC were larger, but a higher number of changes were detected in the direction of HPLC. In comparison to the taxonomy results, very strong ORs were observed for the biggest differences in ortholog groups between diets. Tenfold or greater differences were observed for 45 and 211 ortholog groups with HPLC and MPMC, respectively (average OR of 48.17 and 0.06). Hundredfold or greater ORs with MPMC were observed for 19 ortholog groups (average OR of 202.25).

### Protein-related pathways were ranked highly among pathways differing between diets

To identify the biochemical pathways containing the majority of ortholog groups significantly different by diet, we carried out a permutation analysis. This approach identified 10 out of 194 pathways that contained more significantly different ortholog groups than expected by chance (with *p*<0.022; **Table 2** **, Table S6**). Those 10 pathways contained 263 unique (300 in total, as the same ortholog group can be associated with more than one pathway) ortholog groups with significant diet effects. The over-represented KEGG pathways involved six pathways of amino acid biosynthesis and metabolism and one pathway each from the respective categories: amino acid related enzymes, ribosome biogenesis, terpenoid backbone biosynthesis and flagellar assembly. 198 out of the 300 ortholog groups were more prevalent in MPMC and 102 ortholog groups were more prevalent in HPLC.

**Table 2:** Pathways containing an over-representation of enzyme functions with significant diet differences.

Pathway ID	DB	found	sig	MPMC	HPLC	Pathway name
**ko00300**	40	36	20	16	4	Lys biosynthesis
**ko00400**	62	55	28	24	4	Phe, Tyr and Trp biosynthesis
**ko03009**	327	76	39	27	12	Ribosome Biogenesis
**ko00270**	66	59	30	27	3	Cys and Met metabolism
**ko00620**	73	67	31	14	17	Pyruvate metabolism
**ko00330**	128	109	47	30	17	Arg and Pro metabolism
**ko00250**	55	43	25	20	5	Ala, Asn and Gln metabolism
**ko00900**	35	28	16	12	4	Terpenoid backbone biosynthesis
**ko02040**	41	40	26	0	26	Flagellar assembly
**ko01007**	104	84	38	28	10	Amino acid related enzymes

For each pathway the following numbers of ortholog groups are given: total in the KEGG pathway, identified in feline feces, significantly different between diets, higher prevalence in MPMC and higher prevalence in HPLC.

**Figure 3:**
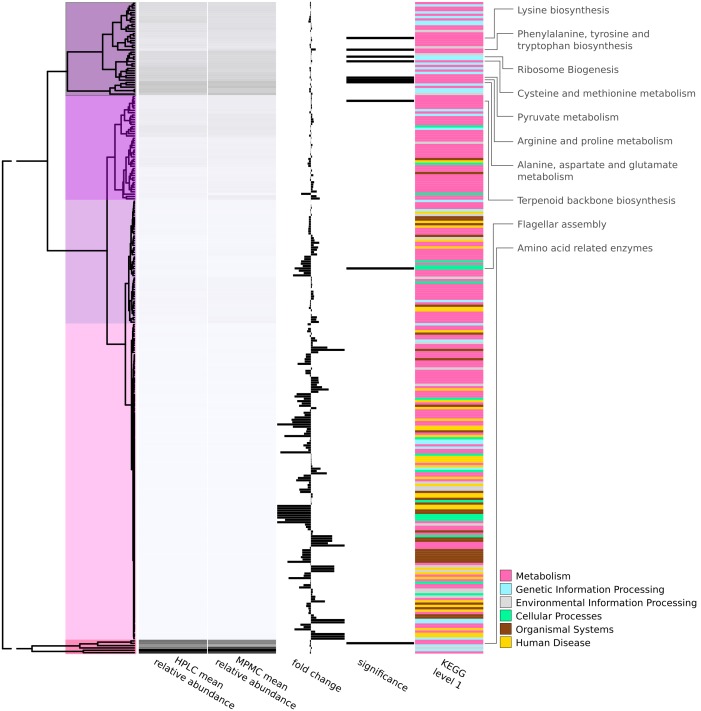
Functional profiles for 280 biochemical pathways (KEGG level 3). Pathways were clustered and sorted by their average relative abundance in the two diets. Cluster structure is indicated by colors overlaid onto the dendogram. Fold changes (full bar equals to a six fold change) to the left indicate a higher abundance in high protein diet (HPLC). The next column indicates ten pathways with an over-representation of ortholog groups (KEGG level 4) with significant diet differences. Six out of ten pathways (names given in right column) are related to amino acid metabolism highlighting the effect of differences in dietary protein on the microbiome.

An overview of all 280 biochemical pathways identified in feline feces in this study and their average prevalence in the MPMC and HPLC microbiomes is given in **Figure 3** (underlying data available in **Table S7**). Mean relative abundance was calculated by adding up the relative abundances of all ortholog groups in the respective pathways (for all samples in the respective diet group). Fold changes between HPLC and MPMC diets were obtained by dividing mean relative abundances of both groups. Clustering based on the abundance patterns revealed five major groups (**Table 3**). The clusters differed with regards to pathway completeness (the number of enzymes identified vs. the number of enzymes in a pathway), average prevalence and fold changes (**Table 3**). On average, the pathways in cluster A contained 84.0 out of 134.5 total enzymes (62.5% completeness). Clusters B, C, D and E were 50.1%, 34.1%, 8.6% and 70.4% complete, respectively.

**Table 3:** Clusters of biochemical pathways identified in feline feces.

	Pws	Ret	DB	Fnd	Ovr	% abu	FC	M	G	E	C	O	H
**A**	40	40	134.5	84.0	7	1.15%	1.14	23	13	4	0	0	0
**B**	45	45	90.8	45.5	1	0.36%	1.27	35	3	1	3	2	1
**C**	53	40	50.66	17.32	1	0.11%	1.56	31 (25)	3 (3)	3 (3)	4 (4)	5 (1)	7 (4)
**D**	136	71	57.23	4.85	0	0.01%	4.03	54 (41)	9 (9)	12 (3)	11 (2)	23 (6)	27 (10)
**E**	6	6	244.7	172.2	1	3.44%	1.14	3	2	1	0	0	0

The following data are provided for each cluster: Pathways per cluster, number of pathways retained after MinPath removal, average number of enzymes per pathway in database, average number of enzymes identified in feline feces, number of over-represented pathways, average pathway abundance, number of pathways from the KEGG top level groups of Metabolism, Genetic Information processing, Environmental Information Processing, Cellular Systems, Organismal Systems and Human disease (numbers in brackets are the number of pathways retained after MinPath removal).

The six pathways of amino acid biosynthesis and metabolism and the ribosome genesis pathway were grouped together in cluster A. The pathways of terpenoid backbone biosynthesis, flagellar assembly and amino acid related enzymes were grouped in clusters B, C and E, respectively (**Table S7**).

The MinPath [20] method was used to identify the minimal set of pathways necessary and sufficient to explain the enzyme functions. There were 78 pathways identified as potential artifacts: 13 from cluster C and 65 from cluster D (**Table 3** and **Table S7**). In cluster C 19% of pathways from the category the KEGG top level category metabolism, 80% from organismal systems and 43% from human disease were potential artifacts. In cluster D 24% of pathways from the category metabolism, 75% from environmental information processing, 82% from cellular systems, 74% from organismal systems and 63% from human disease were potential artifacts.

Based on the data presented in **Table 2**, **Figure 3**, and **Table S4**, the microbiomes of kittens fed MPMC included genes with a greater abundance in pathways associated with the biosynthesis of amino acids, vitamins, fatty acids, and peptidoglycans; the glycolytic, TCA, and pentose phosphate pathways; oxidative phosphorylation; and the metabolism of purines, pyrimidines, and sugars. In contrast, the microbiomes of kittens fed the HPLC diet had greater abundance of genes associated with flagellar assembly, chemotaxis, sporulation, and two-component systems.

### Time-related differences

Significant differences in relative abundances over time were detected for 18 genera, 11 families and 360 ortholog groups with *p*<0.00009, *p*<0.00022 and *p*<0.000007, respectively (**Table S4**). With the exception of one genus (*Parabacteroides*; 2.1%) and the family that genus belongs to (*Porphyromonadaceae*; 3.6%) all taxa concerned had relatively low overall abundances (average relative abundances of 0.2% for genera and 0.4% for families). *Parabacteroides* had decreasing odds with time in addition to increased odds with HPLC. Of the 360 ortholog groups with significant time differences, 56 and 304 had increased and decreased odds with time, respectively. Cumulative relative abundance of all ortholog groups with significant time differences was 0.8%.

## Discussion

Recently, a growing number of 16S rRNA-based taxonomy and shotgun metagenomics studies have started to build our understanding of gut microbiota. Such studies are focused almost exclusively on humans, other omnivores or herbivores. The gut microbiota of cats and obligate carnivores in general are still poorly understood. There is a limited number of taxonomy studies [16], [21]–[23] and only two other shotgun studies examining the potential gene functions of the feline gut microbiome [24], [25]. Moreover, except from the original 16S rRNA study of the same samples analyzed here by shotgun metagenomics [16], no other longitudinal cat study has been published to date. To our knowledge, this is by far the largest and most comprehensive analysis of the feline gut microbiome to date. It provides a broad view of the microbial taxa and gene catalogue present in the cat, and what taxa/functional groups are most impacted by altering the dietary carbohydrate: protein ratio.

### Metagenome annotation through short read binning

When processing a large dataset of shotgun sequences generated on the Illumina platform there is the choice of two fundamentally different approaches; assembly of the short reads into longer contiguous sequences (contigs) or binning them into groups of taxonomy and function. For an overview on the strategies for analyzing shotgun metagenomics data see [26]. Each approach has its strengths and weaknesses, but the literature is inconclusive about what approach is best. For each strategy there is a vast choice of analytical methods and algorithms available. The advantage of contigs is that open reading frames (ORFs) can be identified and longer sequences compared to reference databases thereby increasing annotation accuracy. The disadvantage is that although attempts have been made to modify short read assemblers to perform better for metagenomics datasets (e.g. metavelvet [27], an extension to the widely used velvet assembler [28]), de Brujn graph assemblers assume a clonal genome. Assembly of metagenomics datasets often results in extremely fragmented assemblies and can also lead to chimerical contigs of short reads belonging to two or more species. Also due to the complexity of metagenomes compared to genomes, the memory requirements are very large (in excess of 256 Gigabytes). The issue with binning approaches is that to reach acceptable levels of sensitivity, very strict thresholds have to be applied thus reducing the total number of reads assigned taxonomy or function.

For practical reasons, we decided to use MetaCV [19], a binning approach with similar accuracy to blastx [29], the traditional approach for annotating nucleotide sequences against a protein database, while performing ∼300 times faster [29]. MetaCV is a novel tool that applies a composition vector method to the amino acid space. Such methods are well established for nucleotide-nucleotide comparisons (e.g. S-GSOM [30] or TACOA [31]), but novel for protein sequences.

An average annotation rate of 32% was observed at the genus level despite the increasing number of sequenced microbial genomes (1,691 in reference database used for this study and 2,406 available on NCBI as of April 2013). This indicates that the majority of microbial diversity in feline fecal samples is not accounted for in the reference database of sequenced genomes. Another consequence of the short read binning approach was the high number of taxa or enzyme functions with very low sequence counts. They might represent truly rare taxa or orthologous gene groups. However, it is more likely that they are false positives; a side effect of the very high sequencing depth in this study. In the taxonomy results for example, the method identified 1,114 out of the 1,115 species present in the database.

### Significant effects of dietary protein on large parts of microbiota are mostly consistent with previous studies

To date, only two studies have characterized the taxonomic composition and functional capacity of the feline fecal microbiome using shotgun sequencing [24], [25]. Moreover, few studies have evaluated the effects of diet on the feline fecal microbiome using any high-throughput strategy [16], [21], [25]. Similar to what has been reported in those studies and that of other host species, a few taxa accounted for a majority of sequences in the current study. Firmicutes (46.0–61.8% of sequences) was the predominant bacterial phylum in all kittens, followed by Bacteroidetes (17.1–36.0%), Actinobacteria (4.2–17.7%), Proteobacteria (2.0–5.9%), and Fusobacteria (0.2–2.4%). Although the same five phyla were predominant in the current dataset (using Illumina shotgun sequencing) and that of Hooda et al. [16] whereby 16S rRNA-based primers and 454 pyrosequencing was used, the abundance of Bacteroidetes differed greatly between studies. The 16S rRNA primers used in that study appear to be biased against Bacteroidetes, with only 0.2–0.7% being reported in these same samples [16]. Given the lack of Bacteroidetes reported in that study, the other predominant phyla were represented in greater proportions (71.0–80.1% Firmicutes; 4.6–28.5% Actinobacteria; 0.1–12.9% Fusobacteria; 0.1–3.7% Proteobacteria). Although the proportions between studies differ, Firmicutes, Bacteroidetes, Actinobacteria, Proteobacteria, and Fusobacteria are the consistently predominant phyla in cat feces [21], [24], [25].

Despite the differences in technology used, the changes due to diet were relatively similar between the current study and what has been reported previously [16], [32]. At the phylum level, the current study identified fewer Firmicutes and Actinobacteria, but greater Fusobacteria in HPLC-fed kittens consistent with previous studies [16], [32]. Proteobacteria were also greater in HPLC-fed kittens consistent with Hooda et al. [16] but inconsistent with Bermingham et al. [32] who compared a wet HPLC to a dry MPMC diet. At the genus level, many of the predominant groups and dietary-induced changes were consistent with what has been reported previously [16], [32]. In all studies [16], [32], *Megasphaera* was a predominant taxon and present in much greater proportions in kittens fed MPMC compared to HPLC. *Megasphaera* are predominant and important bacteria in ruminants because of their ability to ferment lactate into several short-chain fatty acids (SCFA), including butyrate [33], [34]. As probiotic treatments, *Megasphaera* have been reported to improve gastrointestinal health of rats [35] and pigs [36]. Because the MPMC diet contained more dietary fiber than the HPLC diet (6.9 vs. 2.0%; [37]), a greater population may be expected. The high abundance of this bacterium in the feline gut, accounting for >30% of sequences at 12 and 16 week of age, however, indicates a major role in an obligate carnivore. A greater research focus on this genus in the future is justified.

*Bifidobacterium* and *Acidaminococcus* were predominant genera and in much greater abundance in MPMC-fed vs. HPLC-fed kittens. This finding is consistent with Hooda et al. [16] but inconsistent with Bermingham et al. [32] who either reported very low abundance or no presence at all. Bifidobacteria are predominant bacteria in human infant feces [38], [39] and are associated with gastrointestinal health in humans [40] and cats [41]. Even though some studies have had difficulty detecting bifidobacteria in cat feces [42], [43], recent studies have verified their presence and ability to increase with the addition of fermentable carbohydrates to the diet [44]–[46].

The current study highlights *Selenomonas* and *Prevotella* as being more abundant in MPMC-fed kittens, taxa not reported as being different between dietary groups previously [16], [32]. In general, selenomonads are metabolically diverse, possessing the ability to use a wide variety of nitrogen- and carbon-based substrates. *Selenomonas ruminatium*, the member of this genus most studied because of its importance in ruminant nutrition, is an important lactate-fermenting organism [47] that grows well in environments with a high lactate and organic acid production [48] and is often more abundant in animals fed fermentable carbohydrate-containing diets [49]. Previously reported diet related differences in *Lactobacillus* (greater in MPMC-fed kittens [16], [32]) were not statistically significant in the current study.

*Fusobacterium*, *Clostridium*, *Eubacterium*, *Ruminococcus, Bacteroides* and *Desulfovibrio* were significantly more prevalent in HPLC vs. MPMC. Our findings for *Fusobacterium* and *Clostridium* are in agreement with both previous studies [16], [32], while our results for *Eubacterium* and *Ruminococcus* are consistent with Hooda et al. [16] and our findings for *Bacteroides* are in agreement with Bermingham et al. [32]. Our findings for *Desulfovibrio*, a sulfate-reducing bacteria and member of the Proteobacteria, have not been described previously. Although members of the genera *Fusobacterium*, *Clostridium*, and *Desulfovibrio* are sometimes associated with gastrointestinal disease [41], [50], [51], the kittens in the current study were healthy throughout the study so their presence was more likely due to their ability to ferment protein-based substrates.

The *Bacteroides* and *Prevotella* shift noted in the current study is consistent with data from humans, with *Bacteroides* being more abundant in those eating high protein and animal fats and *Prevotella* being more abundant in those eating more carbohydrates [52]. Gut bacteria of kittens consuming the MPMC diet would be expected to have more total and carbohydrate-based substrate than those consuming the HPLC diet.

### Significant effects on taxonomy are in agreement with targeted 16S sequencing

Statistical analyses were carried out to identify significant differences due to diet, time, and diet by time interactions similar to that performed on 16S sequence data [16]. This enabled a comparison of two studies using different sequencing approaches on the same fecal samples. Hooda [16] presented statistical results for 18 genera and ten families. Our study included data for ten of those genera and all ten families while eight genera were lacking from our reference database (**Table S8**). Choosing the number of annotated 16S reads as a denominator this allows a comparison based on 56% and 86% of sequences for genera and families, respectively (or 46% and 54% when the number of annotated shotgun reads was chosen). We consider this a very good overlap given the disparity of more than two orders of magnitude between the number of taxa in the Hooda [16] reference database (>300,000 16S bacterial sequences) and the reference database of this study (1,691 fully-sequenced genomes).

Among the ten genera available for comparison, eight had significant diet differences, while no significant associations with time or diet by time interactions were observed (**Table S8**). Hooda et al. [16] also identified significant diet differences for the same eight species, but for two of those (*Acidaminococcus* and *Megasphaera*) they also identified significant time effects and diet by time interactions. They also found a significant time difference for *Lactobacillus*. Out of the ten families available for comparison, we found seven with significant diet differences, one family with diet by time interactions (*Erysipelotrichaceae*) while no significant associations with time were observed (**Table S8**). Hooda et al. [16] identified significant diet differences for six families (all of which are included in our list), but did not find significant diet differences for *Lachnospiraceae*. They also identified one family with significant diet differences (*Lactobacillaceae*) and one family with significant time differences and diet by time interactions (*Erysipelotrichaceae*). In summary, the statistical results were in agreement for seven out of ten genera and seven out of ten families (**Table S8**).

We conclude that where a direct comparison between the 16S and the shotgun sequencing data was possible the statistical analyses generated similar results. This is despite the fact that proportions inferred could be very different between methods. For example, the genus *Ruminococcus* had average percentages of 3.2 in MPMC and 13.1 in HPLC with 16S and 0.4 and 1.2 with shotgun sequencing. Despite those differences, the statistical comparisons found significant diet difference in both studies.

### HPLC microbiota are more diverse and stable

Diversity of the HPLC microbiota was significantly higher but residual variability was lower in comparison with MPMC. In a previous study [32], kittens fed a wet HPLC diet had a more diverse fecal microbiota than those fed a dry MPMC diet. We speculate that the more diverse and less variable taxonomic structure observed for the high protein diet reflects specialized microbiota, while the less diverse and more variable structure on the moderate protein diet reflects more generalized microbiota. The fact that the microbiota of the cats fed HPLC were more similar to each other than those of cats fed MPMC (with shorter branch lengths in the cluster dendogram of **Figure 2** and a tighter 95% confidence interval in **Figure 1a**) could also reflect that the HPLC group consisted of two litters and the MPMC group of four litters. However, this may be unlikely as studies examining the impact of host genetics on the microbiota have been inconclusive and most studies could not identify associations [53].

### Little evidence of microbiome development between 8 and 16 weeks of age

It is known that the microbiome develops early in life [54], however in this eight week period little effect was seen. Only 18 bacterial genera and 11 families showed significant differences in their relative abundances over time and with the exception of the genus *Parabacteroides* and the family *Porphyrmonadaceae* (the family this genus belongs to), all taxa were rare. Those two taxa were also significantly different by diet. No significant differences in Shannon diversity over time were found. 360 ortholog groups had significant differences over time but their cumulative abundance contributed to less than one percent of the annotated sequences. These data can be used to suggest that the microbiome of growing kittens with access to mothers diet, are established by week eight and little changes happen between eight and 16 weeks of age.

Kittens started eating solid cat food at three weeks of age and gradually increased their intake of solid food and decrease nursing until weaned by their mothers (starting at week five). As kittens were housed with their mothers, who were fed the same diets later assigned to the kittens, this exposure could have shaped the kittens’ microbiota from a very early age. Studies in humans have shown the mother’s milk microbiota to be complex [55] and concluded the bacterial composition plays an important role in the colonization of the infant gut [55], [56]. One hypothesis is that evolution has shaped obligate carnivores and their gut microbiota in a way that facilitates a rapid transition from the digestion of mother’s milk to prey.

Although few differences over this relatively short period of time were identified in this study, future longitudinal studies of the feline fecal microbiota may provide further insights into the development and maintenance of microbiota. Studies designed to characterize the development of the gut/fecal microbiota over a longer period of time and into adulthood are needed.

### Gene identification and biochemical pathway inference from metagenomes

The number of unique genes identified in the metagenomes of feline feces was approximately 108-fold higher than the number of open reading frames identified in the cat genome [57]. This ratio is consistent with what was proposed for humans (100-fold; [58]) and later verified experimentally (150-fold; [59]). To understand better the metabolic potential related to diet the genes were mapped on to the KEGG database. Using this approach 55.8% of the genes in the feline feces were mapped to ortholog groups. Whilst this approach excluded proteins without enzymatic function (e.g. structural proteins) it did not affect our central question about diet-induced differences in the functional potential of the microbiomes.

The inference of biochemical pathways does not only depend on the number of genes and ortholog groups identified, but also on how they are mapped to the KEGG reference pathways. A naïve approach that called a pathway present when it had at least one enzyme present identified 280 pathways in the microbiomes of feline feces. This approach is problematic because a single enzyme may function in more than one pathway [60] with “promiscuous enzymes” (enzymes occurring in more than one pathway) resulting in an over-estimation of the number of pathways.

Clustering the pathways based on their average relative abundance highlighted cluster D, a group of 136 pathways with very low relative abundances and low pathway completeness. It is very likely those pathways are false positives caused by enzymes with low sequence counts and promiscuous enzymes. This is also the cluster from which the largest proportion of pathways was removed by MinPath [20].

MinPath [20] reduced the number of pathways from 280 to 202 by identifying promiscuous enzymes. This more conservative estimation affected clusters C (13) and D (65) and removed mostly (in terms of the proportion of pathways removed) pathways related to cellular systems, organismal systems and human disease; all pathways one would not expect to find in the prokaryotic genomes in feline feces analyzed in this study. This shows that most falsely identified pathways (e.g., human disease related) are not the consequence of an erroneous sequence annotation during the MetaCV binning (the reference database contained no eukaryotic sequences), but indeed caused by promiscuous enzymes playing roles in more than one pathway. We recommend that any study working with pathway data inferred by automatic annotation of sequence apply MinPath or a similar approach to reduce the number of false positives.

It is important to note that although the concept of separate biochemical pathways can facilitate understanding, in the cell all pathways are connected to some degree by the enzymes they contain [61]. This makes it difficult to assess in an automated way whether a pathway is functional or not. In some cases, a lot more enzyme functions can also be included into a single pathway than are needed for the pathway to function biochemically. A good example is the DNA replication pathway in which we identified 24 out of 51 enzymes. This would suggest an incomplete and potentially non-functional pathway. Highlighting the enzymes identified in the respective pathway revealed that in the KEGG database prokaryotic and eukaryotic DNA replication are combined into a single pathway and the microbes in feline feces have all the enzymes necessary for prokaryotic DNA replication.

### Strongest diet-related differences between pathways were related to amino acid metabolism

We identified 2,013 enzyme functions that were significantly different by diet. Interpreting all of those functions and the 194 pathways they belong to, in the context of diet, is prohibitive. It also bears the risk of over-interpreting the results before having established the robustness of the inference methods given all their shortcomings (see discussion on methods for annotating shotgun metagenomics data and pathway inference).

Therefore, we first took a top level view at the data and used an objective method to identify the biochemical pathways containing most diet-related changes before addressing any specific changes. Our permutation testing approach identified ten pathways that contained more significant changes in their enzymes than expected by chance (*p*<0.022). Six of those pathways were related to the biosynthesis and metabolism of amino acids. Given that the main difference between the diets fed in this study was their protein content, it is of note that the biggest differences in the metabolic potential of the two fecal microbiomes lie in their ability to metabolize amino acids. This finding is an indication that the fecal microbiomes have functional differences related to the contrasting protein content in the diets and that they may reflect the functional differences of the microbiomes of the GI tract, making the fecal microbiome a suitable surrogate for that of the GI tract. It may also be interpreted as a validation of the bioinformatics and statistics methods applied in this study, as they are robust enough to identify biologically-relevant diet differences.

### Biochemical pathways identified in feline feces are consistent with previous studies

Predominant KEGG functions in the microbiomes of feline feces are summarized in **Table S9** and included replication and repair (HPLC: 12.7% of sequences; MPMC: 12.2% of sequences), amino acid metabolism (HPLC: 12.1%; MPMC: 12.6%), carbohydrate metabolism (HPLC: 11.6%; MPMC: 11.2%), translation (HPLC: 9.1%; MPMC: 7.7%), nucleotide metabolism (HPLC: 7.5%; MPMC: 6.5%), membrane transport (HPLC: 6.6%; MPMC: 7.2%), energy metabolism (HPLC: 6.2%; MPMC: 5.9%), folding, sorting and degradation (HPLC: 3.5%; MPMC: 3.1%), and metabolism of cofactors and vitamins (HPLC: 2.9%; MPMC: 3.6%). These data are similar to that of the previous feline gut metagenomic studies [24], [25], and represent the core functions of a gut microbiome, including carbohydrate and protein enzymatic breakdown and metabolism; energy metabolism, and replication (replication and repair; nucleotide metabolism).

### Diet-related differences in enzyme functions and biochemical pathways

In the current study, the microbiome of MPMC-fed kittens had a greater relative abundance of genes associated with the biosynthesis and metabolism of several amino acids, including alanine, aspartate/asparagine, and glutamate/glutamine; cysteine and methionine; lysine; tyrosine; and phenylalanine, tyrosine, and tryptophan. The abundance of genes associated with the biosynthesis of several vitamins (thiamin, riboflavin, vitamin B6, niacin, biotin, and pantothenate), fatty acids, and peptidoglycans and genes associated with the glycolytic, TCA, and pentose phosphate pathways; oxidative phosphorylation; and the metabolism of purines, pyrimidines, and sugars also were more prevalent in MPMC-fed kittens. Biosynthesis of amino acids and fatty acids are critical for the growth of all microbes, but microbial activity level, the amount and/or profile of amino acids produced, and production of other protein-containing metabolites have been reported to vary depending on dietary composition in pigs [62], cattle [63], and cats [44], [64]. Abundance of urease subunits-α, β, and γ were also greater in MPMC-fed kittens. Urease activity is important for nitrogen recycling, especially when low-protein diets are consumed [65]. Both diets fed in this study provided sufficient dietary protein for growing kittens. However, a greater amount of fermentable carbohydrates and lower amount of dietary nitrogen reaching the large intestine in MPMC-fed kittens would be expected to require greater amino acid biosynthetic and urease capacity and/or activity.

### Potential for mucin degradation is higher in HPLC microbiomes

Mucins are high-molecular-weight glycoproteins that not only form a protective mucus barrier in the gut, but may have a role in regulating bacterial colonization and metabolism [66], [67]. Many bacteria may adhere to mucus, using it as an anchor for colonization, while others have developed an ability to utilize it as a substrate, possessing glycoside hydrolase, sulfatase, fucosidase, or protease enzymes. The abundance of three genes related to mucin degradation, including arylsulfatase (OR 0.21), alpha-L-fucosidase (OR 0.55), and mannosyl-glycoprotein endo-β-N-acetylglucosaminidase (OR 0.27), was greater in HPLC-fed kittens. A fourth, O-sialoglycoprotein endopeptidase, was slightly more abundant (OR 1.35) in MPMC-fed kittens. Even though exogenous carbohydrates are often the focus of gut microbiome metabolism, many taxa have the ability to degrade GI mucins. The difference in this study may be due to the greater presence of *Bacteroides* and lower presence of *Bifidobacterium* in the HPLC-fed kittens. The ability of *Bacteroides* to utilize mucins is well established [68], [69]. In contrast, other than *B. bifidum* that possesses several glycoside hydrolases capable of mucin degradation [70], most *Bifidobacterium* are restricted in that regard [71].

### Summary and conclusion

We carried out shotgun sequencing of DNA isolated from fecal samples of kittens fed a high- and a medium-protein diet to determine the effect of protein content on the taxonomic structure and functional potential of the gut microbiome. We identified 324 genera, 138 families and 2,013 KEGG ortholog groups with significant diet differences. Biochemical pathways related to amino acid metabolism were highly ranked among the pathways containing KEGG ortholog groups with diet differences. This result links differences in protein content in the hosts’ diet with differences in the functional potential of the microbiomes to metabolize protein. It also suggests that feces can be used as a surrogate for the gut in comparative analyses of microbiomes, and that short read binning approaches is able to detect those differences. The naïve approach of pathway identification greatly overestimated the number of pathways in feline feces and methods like MinPath need to be applied to give more conservative estimates. Very few taxa and KEGG orthology groups had significant differences over time between weeks 8 and 16, but more studies investigating changes further into adulthood are needed.

## Materials and Methods

### Animals and experimental design

The animal protocol was approved by the University of Illinois Animal Care and Use Committee and was conducted at the Edward R. Madigan Laboratory of the University of Illinois. Eight domestic shorthair female cats [3.5±0.24 kg body weight (BW) and 1.3±0.02 yr of age] and one male domestic shorthair cat (5.8 kg; 1.3 yr of age) were parents to these kittens. Female cats were randomly assigned to two test diets, one month before mating, and continued throughout gestation and lactation. Kittens from mothers fed the MPMC diet (n = 6) or HPLC diet (n = 6) were studied herein. Kittens were housed with dams until eight weeks of age, weaned and then fed the same diets as mothers. After weaning, kittens were twin- or triple-housed within dietary group in cages (1×0.76×0.7 m) in temperature-controlled rooms with ad libitum food and water intake to allow for adequate growth. Fresh fecal samples (within 15 min of defecation) were collected from all kittens at 8, 12, and 16 weeks of age. All fecal samples were stored immediately at −80°C.

### Diets

Complete dietary information is provided in [37]. Briefly, both diets were formulated to meet or exceed all nutrient requirements for growth according to the Association of American Feed Controls Officials [72] and were based on chicken meal, dried potato product, chicken fat, dried egg, herring meal, and beet pulp. The MPMC diet contained approximately 78 g crude protein and 44 g fat/1000 kcal ME (dry matter basis), while the HPLC diet contained approximately 110 g crude protein and 49 g fat/1000 kcal ME (dry matter basis).

### DNA Extraction and Illumina Sequencing

Bacterial DNA was extracted using a QIAamp DNA stool mini kit (Qiagen, Valencia, CA, USA) using the repeated bead beating plus column (RBB+C) method [73]. Fecal DNA was quantified using a NanoDrop ND-1000 spectrophotometer (NanoDrop Technologies, Wilmington, DE, USA). DNA quality was assessed before Illumina sequencing using a 2100 Bioanalyzer (Agilent Technologies, Santa Clara, CA). Illumina sequencing was performed at the W. M. Keck Center for Biotechnology at the University of Illinois using an Illumina HiSeq2000 sequencer (Illumina Inc., San Diego, CA) according to manufacturer’s instructions. Briefly, DNAseq libraries were first prepared with Illumina's TruSeq DNAseq Sample Prep Kit. The libraries were quantified by qPCR, pooled in groups of 3, and each pool was sequenced on one lane for 100 cycles from each end of the fragments on a HiSeq2000 using a TruSeq SBS Sequencing Kit (version 3) and analyzed with Casava1.8 (pipeline 1.9). Sequence reads for all 36 samples were deposited to the European Nucleotide Archive (ENA) under the project accession PRJEB4391 (sample accessions ERS329791–ERS329826).

### Sequence data annotation

Sequencing reads were trimmed using sickle (https://github.com/najoshi/sickle.git) version 1.200 with cutoff of 20 and 50 for phred score and length, respectively. Trimmed short reads were annotated using a composition vector approach as implemented in MetaCV version 0.226 [19] against a reference database of 5,488,681 genes from 1,691 fully-sequenced genomes (supplied with MetaCV). MetaCV results files contain the following data entries for each individual short read in the FASTQ files of the respective samples: Read identifier, quality score of the annotation, GI number of the best protein match, KEGG entry identifier (if available), eggNOG identifier (if available), NCBI taxon ID and strain name. MetaCV applies a lowest common ancestor (LCA) approach on the NCBI taxonomy tree when reliable annotation down to strain level is not possible. Results were filtered by a quality score of 20 (as recommended in the original publication and during personal communication with the authors for read lengths of 100 nucleotides) and collated into a summary table using MetaCV. Summary tables are automatically generated for the following levels of taxonomical hierarchy: superkingdom, phylum, class, order, family and genus. A summary table on the species level (which is required for the calculation of species diversity) was generated separately by parsing the results files. In terms of functional annotation, summary tables were automatically generated for KEGG [17] orthology groups (KEGG level 4), biochemical pathways (KEGG level 3) and the higher level groupings KEGG level 2 and 1, as well as eggNOG [74] gene clusters. Uniprot tables of April 2012 were used to relate genes to KEGG orthology groups and eggNOG clusters. KEGG tables of May 2011 were used to relate ortholog groups to pathways and higher level groupings.

### Statistical analyses

Individual genera, families and ortholog groups were analyzed univariately by generalized estimating equations, with a binomial distribution (for proportional responses) and a logit-link, to investigate the diet effects over time. Kitten was specified as a random effect to account for the lack of independence in observations over time within kittens and diet, week and their interaction were fitted as categorical fixed effects. If effects were found to be non-significant they were dropped from the model. All effects were tested against a data set type I error rate of 5% by Sidak [75] adjustment, thus the critical *p*-values used were 0.000089 for genera, 0.000223 for families and 0.000007 for ortholog groups. Prior to fitting of the univariate model sets, potential responses were filtered if the proportion of zero counts was greater than 25%. All odds ratios (OR) reported were significant at the respective adjusted *p*-value cutoff.

Permutation testing was performed to identify pathways containing more ortholog groups with significant diet changes than would be expected by chance. The number of ortholog groups in each KEGG pathway and the subset with significant diet changes was calculated. One thousand random subsets of 2,013 ortholog groups were then taken (to represent random significant ortholog groups) and the number found in each pathway calculated. The probability of a pathway containing more significant ortholog groups than would be expected by chance was calculated as the percentage of subsets where the random number in each pathway was greater or equal to the number of significant ortholog groups in each pathway. A Sidak [75] adjusted critical *p*-value of 0.002 was used to maintain a 5% type 1 error rate for the 228 pathways tested.

Diversity was estimated by calculation of Shannon indices [76] from the normalized species counts for each sample according to the formula: 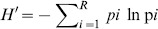, where R is the number of species observed and p_i_ is the proportion of the i-th species. These were then analyzed by linear mixed models with kitten as a random effect, with weighting by diet specific variability, and diet, time and their interaction as categorical fixed effects. Fixed effects were tested against a cut-off of *p*<0.05 and dropped from the model if non-significant. In addition, the heterogeneity of variance between diets was tested by likelihood ratio tests for nested random effect models. Shannon indices of individual samples and the results of the statistical analysis are provided in **Table S3**.

Species richness and diversity were also estimated by calculation of rarefaction curves for each sample. Random permutations were generated by using the Linux command *shuf* on the MetaCV results files. To reduce noise a higher QC cutoff of 40 was applied. Sub-samples were taken at depths of 1, 5, 10, 20, 40 and 60 million reads (unpaired) with ten replicates per depth and sample (2160 data points in total). These were then analyzed by linear mixed models with time nested in kitten as the random effects, with variability weighting by the number of sequences. Diet, time, the number of sequences and their interactions were fitted as categorical fixed effects. Effects were tested against a cut-off of *p*<0.05 and dropped from the model if non-significant. The number of species per sub-sample and the results of the statistical analysis are reported in **Table S3**.

#### Taxonomic profiles (Figure 2)

Raw genus abundance data as inferred by MetaCV [19] was converted to relative abundances using the number of annotated sequence reads as a denominator on a per-sample basis. The 19 most abundant genera irrespective of diet were used for a stacked per-sample bar plot with the remaining genera pooled as ‘others’. The samples were ordered as inferred by the following clustering approach. Clustering was performed on the relative abundances of all genera using R. Pair-wise distances were calculated using Euclidean distances. Hierarchal clustering was performed according to the average agglomeration method which was previously shown to perform best on taxonomic profiles of environmental data [77].

#### Functional profiles (Figure 3)

Raw pathway abundance data (KEGG level 3) as inferred by MetaCV [19] was converted to relative abundances using the number of annotated sequence reads as a denominator on a per-sample basis. Mean relative abundances were calculated and plotted across all HPLC and MPMC samples, respectively. Clustering was performed on mean relative abundances for HPLC and MPMC using Euclidean distance with hierarchal clustering and average agglomeration in R. Fold change was calculated by dividing mean relative abundances of HPLC and MPMC using the smaller value as denominator and plotting the values in the respective direction. The axis of the graph illustrates a minimum fold change of 1 up to a maximum fold change of 6.

## Supporting Information

Table S1 **Total taxonomy and functional assignment counts and percentages of fecal samples from kittens fed a high-protein, low-carbohydrate or moderate-protein, moderate-carbohydrate diet.**
(XLSX)

Table S2 **Taxonomic annotation counts and proportions for superkingdom, phylum, class, order, family, genus, and species of fecal samples from kittens fed a high-protein, low-carbohydrate or moderate-protein, moderate-carbohydrate diet.**
(XLSX)

Table S3 **Shannon diversity indices and rarefaction estimates of fecal samples from kittens fed a high-protein, low-carbohydrate or moderate-protein, moderate-carbohydrate diet.**
(XLSX)

Table S4 **All significantly different genera, families, and KEGG functional units of fecal samples from kittens fed a high-protein, low-carbohydrate or moderate-protein, moderate-carbohydrate diet.**
(XLSX)

Table S5 **KEGG functional unit counts and proportions of fecal samples from kittens fed a high-protein, low-carbohydrate or moderate-protein, moderate-carbohydrate diet.**
(XLSX)

Table S6 **Over-represented KEGG pathways of fecal samples from kittens fed a high-protein, low-carbohydrate or moderate-protein, moderate-carbohydrate diet.**
(XLSX)

Table S7 **Biochemical pathways identified in feces kittens fed a high-protein, low-carbohydrate or moderate-protein, moderate-carbohydrate diet.**
(XLSX)

Table S8 **Comparison of shotgun- and 16S-based taxonomic annotation proportions for family and genus of fecal samples from kittens fed a high-protein, low-carbohydrate or moderate-protein, moderate-carbohydrate diet.**
(XLSX)

Table S9 **Predominant KEGG biochemical pathways of fecal samples from kittens fed a high-protein, low-carbohydrate or moderate-protein, moderate-carbohydrate diet.**
(XLSX)
